# Damage to the orbicularis oculi muscle may impair the development of dermatochalasis

**DOI:** 10.1038/s41598-024-52955-y

**Published:** 2024-01-29

**Authors:** Larysa Krajewska-Węglewicz, Paulina Felczak, Dariusz Adamek, Małgorzata Dorobek

**Affiliations:** 1 Department of Ophthalmology, National Institute of Medicine of the Ministry of Interior and Administration, Wołoska 137, 02-507 Warsaw, Poland; 2 Department of Neuropathology, Institute of Psychiatry and Neurology, Warsaw, Poland; 3 Department of Pathomorphology, Jagiellonian University Medical College, Cracow, Poland; 4 Department of Neurology, National Institute of Medicine of the Ministry of Interior and Administration, Warsaw, Poland

**Keywords:** Eyelid diseases, Eye abnormalities

## Abstract

The purpose of this article is to investigate the changes that occur in the orbicularis oculi muscle (OOM) in patients with dermatochalasis. The OOM specimens from 26 patients were collected during upper eyelid blepharoplasty. Each specimen was divided into three parts, which were then examined using different techniques: formalin embedding for light microscopy, free freezing for histochemical examination, and fixation in 3% glutaraldehyde for electron microscopy. The severity of dermatochalasis was classified according to the anatomical landmarks. 78 specimens from patients with dermatochalasis were evaluated. Under light microscopy, specimens showed an increase in muscle fiber size variation, rounding of muscle fibers, and lobulation of myocytes in a fibrotic background. Under electron microscopy, loss of myofilaments, vacuolar vesicles, and swollen mitochondria were observed, along with osmophilic aggregates resembling nemadine bodies and collagen fibrils. A statistically significant association between the progression of dermatochalasis and the presence of aggregates resembling nemaline bodies was found (p- value < 0.005). Significant changes occur in the OOM in patients with dermatochalasis and the presence of aggregates resembling nemaline bodies is correlated with the degree of eyelid drooping. Thus, OOM may contribute in dermatochalasis progression.

## Introduction

Dermatochalasis refers to excess skin on the upper eyelid that can cause aesthetic problems and obstruction in the visual field as the skin progressively weakens due to aging, gravity, edema and inflammation^1,2^. The condition is further worsened by a loss of elasticity in eyelid tissues^3^, downward displacement of eyebrows, atrophy of adipose tissue, and muscle descent. Although the loss of skin elasticity is a known factor in dermatochalasis, the influence of histological changes in the orbicularis oculi muscle (OOM) is not clear. The OOM plays a critical role in maintaining healthy vision and the ocular surface, but there is limited research on its morphology and its relation to dermatochalasis.

The gold standard treatment for dermatochalasis is upper eyelid blepharoplasty, which involves removing excess skin and underlying OOM tissue. However, recent studies suggest that OOM preservation may yield better functional and aesthetic outcomes^4–6^. It is difficult to determine when OOM resection is appropriate without understanding the morphological changes in the muscle that contribute to dermatochalasis.

### The aim

The purpose of this study was to examine the morphological changes in the OOM using light and transmission electron microscopes in patients with dermatochalasis and to compare the structural changes in the OOM with the severity of eyelid drooping.

## Methods

The study enrolled 26 consecutive patients (25 women and 1 man) with a clinical diagnosis of dermatochalasis/blepharochalasis, aged 37 to 78 years. All of them underwent upper eyelid blepharoplasty, which involved removal of the skin and OOM. The degree of upper eyelid sagging was graded using a classification system developed by Jacobs et al., which grouped eyelid drooping into four categories: normal (1), mild (2), moderate (3), and severe (4)^7^.

Biological material for histological, histochemical, and ultrastructural research was obtained from muscle biopsies taken from a randomly selected site on the upper eyelid during the upper blepharoplasty procedure.

Each muscle biopsy was divided into three sections. The first section was fixed in 10% neutral buffered formalin, processed, and stained using various histological stains, including hematoxylin and eosin, Gomori trichrome, Congo red stain, Periodic acid Schiff (PAS), and desmin expression. Light microscopy was used to examine the stained slides.

The second section was snap-frozen in liquid nitrogen and then stained using several different techniques, including Nicotine adenine dinucleotide dehydrogenase tetrazolium reductase (NADH), Myosinadenosine triphosphatase (ATPase) at pH levels 9.4, 4.6, and 4.3, Lactate dehydrogenase, and Succinate dehydrogenase.

For electron microscopic evaluation, small fragments of tissues were fixed in 2.5% glutaraldehyde solution, postfixed in 1% osmium tetroxide solution, dehydrated in a graded ethanol alcohol series and propylene oxide, and embedded in Spurr resin. Semithin sections were stained with toluidine blue to select appropriate areas. Ultrathin sections were contrasted with uranyl acetate and lead citrate and then examined and photographed with a transmission electron microscope (TEM), JEOL model 140.

Kruskal–Wallis test was used to describe associations between the severity of OOM damage and the progression of dermatochalasis.

### Ethics approval

This study involves human participants and was approved by the Central Clinical Hospital of the Ministry of Interior and Administration Ethics Committee. All participants were treated in accordance with the tenets of the Declaration of Helsinki. Participants gave informed consent to participate in the study before taking part.

## Results

During the blepharoplasty procedure, a total of 78 samples of the OOM were collected from 26 patients with dermatochalasis. Table 1 depicts the distribution of patients in each group of eyelid drooping.

**Table 1:** Distribution of patients by grades of dermatochalasis.

	n	%
2	7	27
3	9	34.6
4	10	38.4
Total	26	100

n = number of patients, % = group percentage.

### Histological analysis

**Figure 1:**
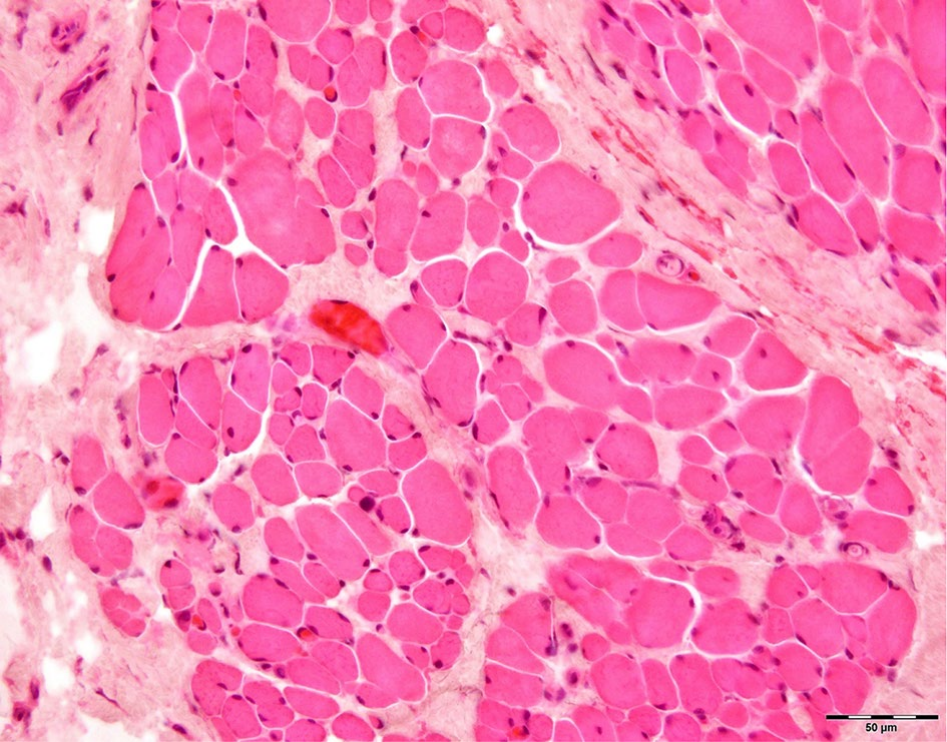
Representative image of OOM hematoxylin and eosin staining showing various sized fibers, round fibers, connective tissue between muscle bundles (Patient No 14, aged 46).

To assess the size, shape, and structural alterations of the muscle fibers in the OOM, we used cross sections of the full thickness of the muscle. Our observations revealed that the OOM fibers were relatively small. The myofibrils formed distinct bundles with fibers oriented in the same directions. The fibers were arranged in parallel elongated, oval, or fusiform fascicles and were relatively loosely packed, with various sizes and mostly rounded shapes. We observed a blurred myofilament structure and noted extensive connective tissue between muscle fascicles, which included an increased amount of endomysial collagen and areas of fibrosis between muscle bundles. The fibers contained a small amount of sarcoplasm, multiple nuclei located laterally, and abundant myofibrils. The mitochondria were moderate in number and distributed uniformly in the sarcoplasm between the myofibrils and in the peripheral zone. Intrafascicular fat cells were also present. Figure 1 illustrates representative case.

### Histochemical analysis

**Figure 2:**
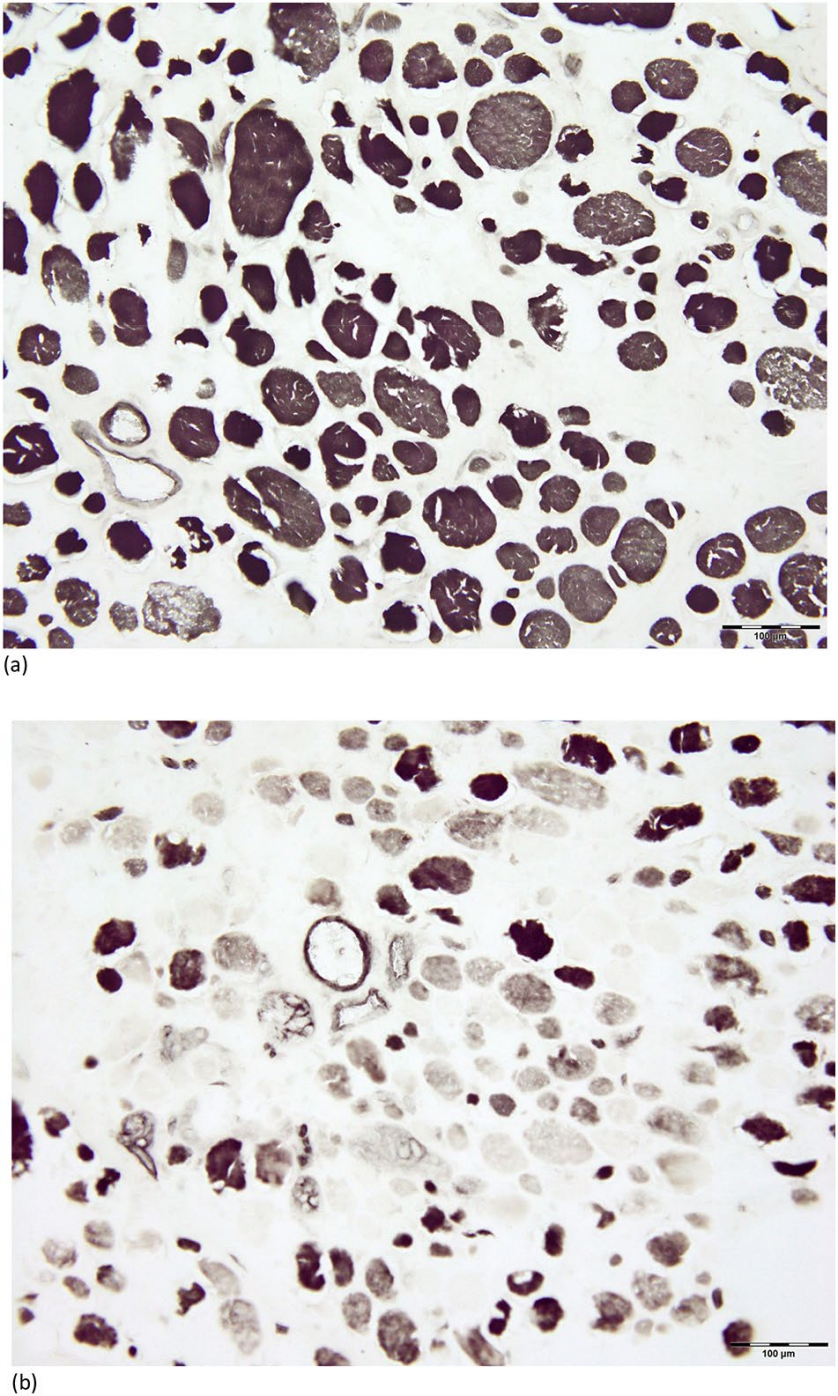
Type II fibers of irregular shapes and different diameters predominance upon staining for ATPase activity at pH 9.4 (**a**) and pH 4.3 (**b**) in patient aged 56 [No. 4].

Histochemical evaluation was performed to identify fiber types using ATPase and NADH-TR staining. The ATPase staining showed that the type II fibers were the predominant type, as they were more numerous, larger, and presented more variation in size than type I fibers (Fig. 2). Type I fibers were less numerous and smaller. The NADH-TR reactions revealed that smaller fibers had a higher oxidative enzyme activity, staining darkly, while larger fibers showed pale staining. Lobulated fibers in varying numbers were also found.

### Ultrastructural analysis

**Figure 3:**
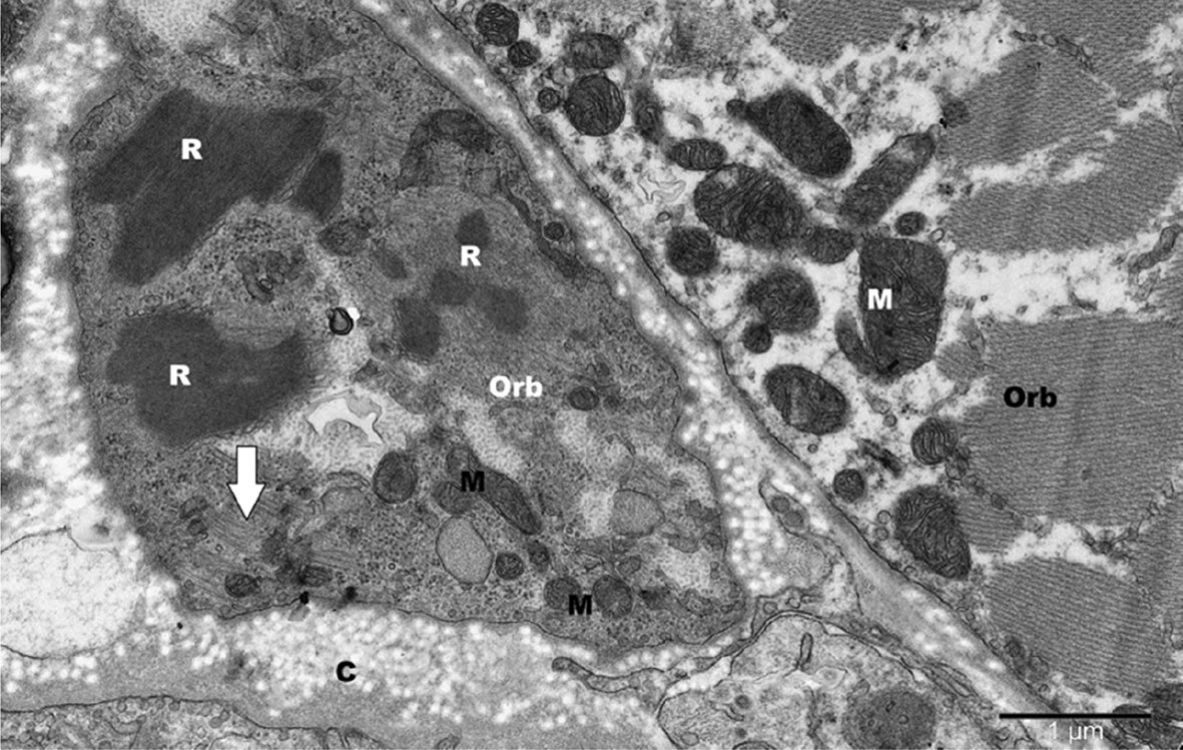
Dark aggregates resembling nemadine bodies (R) is in deformed orbicular muscle (Orb) in patient aged 37 years [No. 26]. The remains of the preserved myofilaments (arrow) in the sarcoplasm are visible. In the adjacent orbicular muscle with the loss of myofilaments, large, malformed mitochondria (M) are located. *C* collagen fibrils. Orign. magn. × 25,000.

The ultrastructural observations using electron microscopy showed varying levels of damage in the OOM of all examined patients, with mild damage in the form of disorganized Z-lines and vacuoles in some mitochondria. The local loss of myofilaments and atrophy, glycogen granules filling empty regions, various-sized vacuolar vesicles and swollen mitochondria, atrophic muscles with blurred myofilament structure, and dark aggregates resembling nemaline bodies between damaged myofilaments were present (Fig. 3).

We found significant correlation between the severity of OOM damage and the presence of aggregates resembling nemaline bodies (Spearman's Rho coefficient, p-value equal 0.02963).

## Discussion

The etiopathogenesis of dermatochalasis is complex, with age, male sex, higher BMI, fair skin color, and smoking being identified as non-genetic risk factors^7^. We decided to investigate the OOM alterations according to the severity of eyelid drooping. Comparison of the damage to the OOM with the degree of dermatochalasis is the most objective assessment. Age is not the only factor responsible for dermatochalasis occurrence and younger patients may present with more severe lid sagging than older ones. Sun exposure is a proven factor in the loss of elasticity of the skin leading to dermatochalasis but OOM is not exposed to sun damage. Therefore we decided not to compare the damage of the OOM with age or skin specimens.

The conventional approach to treating dermatochalasis is upper eyelid blepharoplasty, a procedure that involves the removal of excess skin and underlying OOM tissue. Nevertheless, recent research suggests that preserving the OOM may lead to improved functional and aesthetic outcomes. Thus, elucidating the role of the orbicularis muscle in dermatochalasis development may offer valuable insights into whether sparing the muscle during blepharoplasty is advisable.

It has been shown in previous studies, that the OOM is distinct from extraocular muscles, skeletal muscles, and facial muscles^8^. Human muscles, with the exclusion of small highly specialized muscles and the EOM, are a combination of Type I (slow contracting) and Type II (fast contracting) muscles. Type I fibers are aerobic and high in oxidative activity but low in glycolytic enzyme activity and capable of long continuous activity. Type IIA fibers are both aerobic and anaerobic, contain much glycogen, and are fast contracting but also capable of sustained activity. The fast but not fatigue-resistant Type IIA fibers serve well in blinking. Type IIB fibers are anaerobic with moderate amounts of glycogen and fast contracting. Fast and capable of sustained activity Type IIB fibers are suited for closing the eyes. Our results are consistent with previous studies and show the predominance of Type II fibers in OOM^8–10^. With facial muscles OOM shares similarities like smaller fiber size than limb muscles or greater variation in fiber size. The presence of inclusion bodies in OOM that we found, demonstrates a distinct structural difference in ultrastructural pathology from limb skeletal muscles. The presence of damaged myofibrils and malformed mitochondria, among others, with abnormal cristae or inclusion bodies, indicates the diversity of disturbances ultrastructure of the examined OOM. We observed statistically significant association between the presence of dark aggregates resembling nemadine bodies and the degree of eyelid drooping. Bardosi found the inclusion bodies in the denervated OOM, but pointed out that the inclusion bodies are also present in the aging extraocular muscles^11^. In our specimens, the inclusion bodies were present regardless the age. This is the first study that correlated the presence of aggregates resembling nemaline bodies with the degree of eyelid drooping. Further studies are needed to determine the judgement criteria suitable for clinical practice.

We found various degrees of alterations in all examined samples. Our specimens showed increased variation of muscle-fiber size, rounding of muscle fibers, lobulation, and increased amount of endomysial connective tissue. These features are typically interpreted as myopathic or dystrophic in limb muscles. Previous studies have also reported similar alterations in the OOM, suggesting that these may be the features of normal aging or involutional changes. Choi et al. compared specimens from patients with ptosis and patients with dermatochalasis and found similar to our results histologic, histochemical, and genetic changes in mitochondria in the OOM^12^. They found variation in muscle fiber size, ragged red fibers, and subsarcolemmal accumulation of NADH and SDH in patients with dermatochalasis. They argued that those changes could be attributed to the involutional changes, not myopathy. Similar conclusions had Radnót et al. on the ultrastructural level^13^. McKelvie based on immunohistochemical studies demonstrated that OOM in dermatochalasis resembles mitochondrial cytopathy in limb muscle in patients over 40 years old^14^. Nelson reported comparable alterations in children’s OOM^15^, which may suggest that those are the features of normal OOM. Moreover, it seems that features suggesting atrophy and degeneration in the OOM occur not only in dermatochalasis, since it was also found in ectropion and entropion^16–18^. While Damasceno et al. observed a decrease of elastic fibers in ectropion and entropion^19^, our specimens contained extensive connective tissue.

OOM is divided into orbital and pretarsal parts with the latter further divided into pretarsal and preseptal portions. In our study, all the biopsies were collected from the preseptal part of the OOM. We observed extensive connective tissue between muscle fascicles. The high concentration of collagen in the preseptal OOM remains unclear. Cumming et al. estimated that striated muscles contain a mean of 12% collagen tissue and any excess of connective tissue should be considered pathological^20^. However, Costin et al. showed on cadavers’ OOM that preseptal OOM is composed of 31.5% fibrous tissue and presumed that a higher percentage of connective tissue may be due to the septal fibers investing the muscle^21^. The other explanation is that connective tissue provides stiffness to the unsupported part of the OOM. However, extensive collagen bundles between muscle fibers may be considered pathological and cause malfunction of the OOM. Further investigation is needed to determine the significance of connective tissue background for muscle function.

To our knowledge, this study contains the largest group of ultrastructural examinations of the OOM and adds respective electron microscopic findings to the previously published observations on the OOM. Our findings highlight the need for further investigation into the changes that occur in this muscle. It would be interesting to investigate whether the OOM changes occur predominantly in dermatochalasis or are present in healthy eyelids.

There are several limitations to this study. Ethical considerations prevented us from comparing the OOM specimens of patients with dermatochalasis to those of healthy controls or children. Additionally, we did not perform a gender comparison due to the unbalanced distribution of male and female participants. Moreover, all samples were collected under local anesthesia, which when combined with epinephrine is known to decrease phosphorylase activity and deplete glycogen. Finally, analyzing the association between the OOM damage and its functional implications was beyond the scope of this study.

## Conclusion

Aggregates resembling nemaline bodies occur in dermatochalasis and are associated with the severity of eyelid drooping. Moreover, various degree of muscle alterations are present in dermatochalasis. Consequently, damage to the OOM may impair the development of dermatochalasis but further studies are needed to fully determine it.

## Data Availability

All data relevant to the study are included in the article.
